# Retinal shadows of multisegment lenses in fundus imaging

**DOI:** 10.1111/opo.13524

**Published:** 2025-05-14

**Authors:** Arthur Ho, Lisa Feng, Jos J. Rozema, David A. Atchison

**Affiliations:** ^1^ School of Optometry and Vision Science University of New South Wales Sydney New South Wales Australia; ^2^ Brien Holden Vision Institute Sydney New South Wales Australia; ^3^ Visual Optics Lab Antwerp (VOLANTIS), Faculty of Medicine and Health Sciences Antwerp University Antwerp Belgium; ^4^ Centre for Vision and Eye Research Queensland University of Technology Brisbane Queensland Australia

**Keywords:** broad line fundus imaging, myopia control spectacles, ray tracing, retinal images, retinal shadows, scanning laser ophthalmoscope

## Abstract

**Purpose:**

To provide a more sophisticated explanation of the optics involved when retinal ‘shadows’ are seen in scanning laser ophthalmoscopic images during the wear of multisegment and diffusion optic spectacle lenses.

**Methods:**

Images were recorded with a system that uses a scanning broad line fundus imaging principle in participants with undilated pupils wearing a multisegment spectacle lens. The live infra‐red preview display of the system was also acquired during image recording. Ray‐tracing and image simulations were performed, assuming a Maxwellian illumination system in which a source was refracted first through a lens and then through a model of a multisegment spectacle lens focused onto the pupil of an eye model and hence to the retina. A detector surface was positioned slightly in front of the retina to record the irradiation distribution. The light source was varied from 0.1 μm to 1.8 mm in diameter to investigate the effect of light source size on retinal irradiation distribution.

**Results:**

The retinal shadow pattern was visible on the live infra‐red preview display, as reported previously. However, the recorded images of the retina do not exhibit the same shadow pattern. The simulations predict that the circular shadows corresponding to lenslet positions become progressively less discernible with increasing light source size.

**Conclusion:**

An explanation is provided for the shadows on retinal images due to multisegment lenses, which may be observable under certain illumination conditions.

**Key points**

- Retinal shadows due to multisegment spectacle lenses, previously observed on scanning laser ophthalmoscopic images, are present on the live preview image of a broad line fundus imaging system but not on its recorded image.
- The shadows are explained in terms of the differential refraction of the scanning beam passing through the lenslets and the base lens in the imaging system.
- Simulations predict that circular shadows corresponding to lenslet positions become progressively less discernible with increasing light source size or beam width.

## INTRODUCTION

New spectacle lenses have been developed in recent years for the treatment of myopia progression.^1^ Multisegment lenses contain a central distance region of about 10 mm in diameter, surrounded by a zone in which there is an array of positively powered lenslets on the front surface. Diffusion optics lenses also contain a central distance region, but instead of lenslets, the surrounding zone contains small scattering regions to reduce the contrast on the retina. Such lenses have enjoyed considerable success in slowing myopia progression in children.^1^

de Tomas et al.^2^ were able to image retinal ‘shadows’ caused by different multisegment and diffusion optic lenses using the scanning laser ophthalmoscope (SLO) retina imaging system integrated into an optical coherence tomographic instrument. The magnification of the lenslets onto the retina, for a vertex distance of 12 mm, was calculated to be −0.57, consistent with the incoming beam converging to a position 31 mm beyond the front of the eye. The purposes of this paper were to confirm the reproducibility of such retinal shadows on another imaging system and to acquire a more sophisticated explanation of the imaging and magnification involved in the formation of the retinal shadows.

## METHOD

### Retinal imaging

This study adhered to the Declaration of Helsinki for experimentation on human subjects. Following a protocol (iRECS7185) approved by the institutional ethics committee of the University of New South Wales, one randomly selected eye of two participants was imaged without pupil dilation using a non‐mydriatic retinal imaging system (Clarus 500, Carl Zeiss Meditec AG, zeiss.com/meditec‐ag) while wearing a multisegment, lenslet‐based spectacle lens (−0.50 D, MiyoSmart, Hoya, hoyavision.com). The system uses ‘broad line fundus imaging’ (BLFI), the manufacturer's proprietary technology akin to an SLO but scanning a slit source during image recording.^3^ In addition to the BLFI recorded image from the imaging system, screenshots of the live infra‐red preview window on the computer console were captured during imaging. Computer display screenshots were cropped to the fundus preview window only.

### Ray tracing

The distribution of irradiation on the retinal surface was explored using a computational ray‐tracing model based on that described by Atchison et al.^4^, ^5^ The model was constructed in pure non‐sequential mode in Ansys Zemax OpticStudio (64‐bit, version 2024 R1.00, Ansys, ansys.com).

**FIGURE 1:**
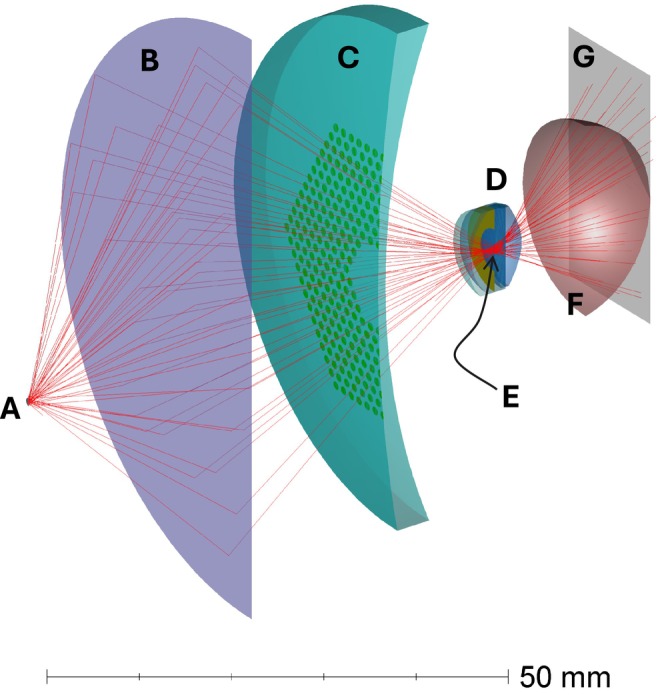
Half‐section of a three‐dimensional (3D) shaded model of the optical layout used in this study showing (A) the circular light source, (B) ideal thin lens, (C) multisegment spectacle lens, (D) Atchison eye model, (E) focus of light source at the plane of the pupil, (F) retina surface and (G) detector. While 1 × 10^8^ rays were traced during analyses, for clarity, only 40 rays are shown here.

Figure 1 shows a three‐dimensional (3D) layout of the model. A model of a multisegment spectacle lens (MiyoSmart) having −4.0 D power^4^ was placed at a vertex distance of 12.0 mm in front of the Atchison myopic eye model,^5^ set to a refractive error of −4.0 D.

To simulate Maxwellian illumination, equivalent to the cumulation of all scanning beam positions over the full field of a retinal scanning imaging system, a light source was focused by an ideal thin lens (Ansys Zemax OpticStudio ‘paraxial surface’) through the spectacle lens to the pupil plane of the eye model. The ideal thin lens is a theoretical zero‐thickness lens producing aberration‐free refraction for rays at all pupil heights and field angles. The light source was modelled with a source referenced to a circular plane surface that emits into a hemisphere with equal probability in all directions. The ideal thin lens had a focal length of 15.0 mm and was placed 30.0 mm towards the eye model from the light source. Focusing of the image of the light source to the pupil plane was achieved by varying the distance between the ideal thin lens and the eye model until a minimum root mean square spot diameter was attained at the pupil plane.

A rectangular plane detector surface was positioned at the retina to record the irradiation distribution. This arrangement differs from an actual retinal imaging system that projects the spherical retinal surface onto a plane image through the nodal point of the combined optical system of the eye and detector. With the plane detector located at the retina, the retinal surface is projected through the aperture stop of the eye. There may be differences in distortion of the image as a result, but this arrangement would not interfere with the primary aim of exploring the formation of retinal shadows.

To explore the effect of light source size on retinal irradiation distribution, the diameter of the light source was varied from 0.2 to 1.8 mm in 0.4 mm steps. In addition, a 0.1 μm diameter source was computed to evaluate an approximate point‐spread distribution.

For each source diameter, 1 × 10^8^ rays were traced through the model and the irradiation distribution at the retina detector was output as a 1024 × 1024 pixel 8‐bit grey‐scale intensity map.

## RESULTS

### Retinal imaging

**FIGURE 2:**
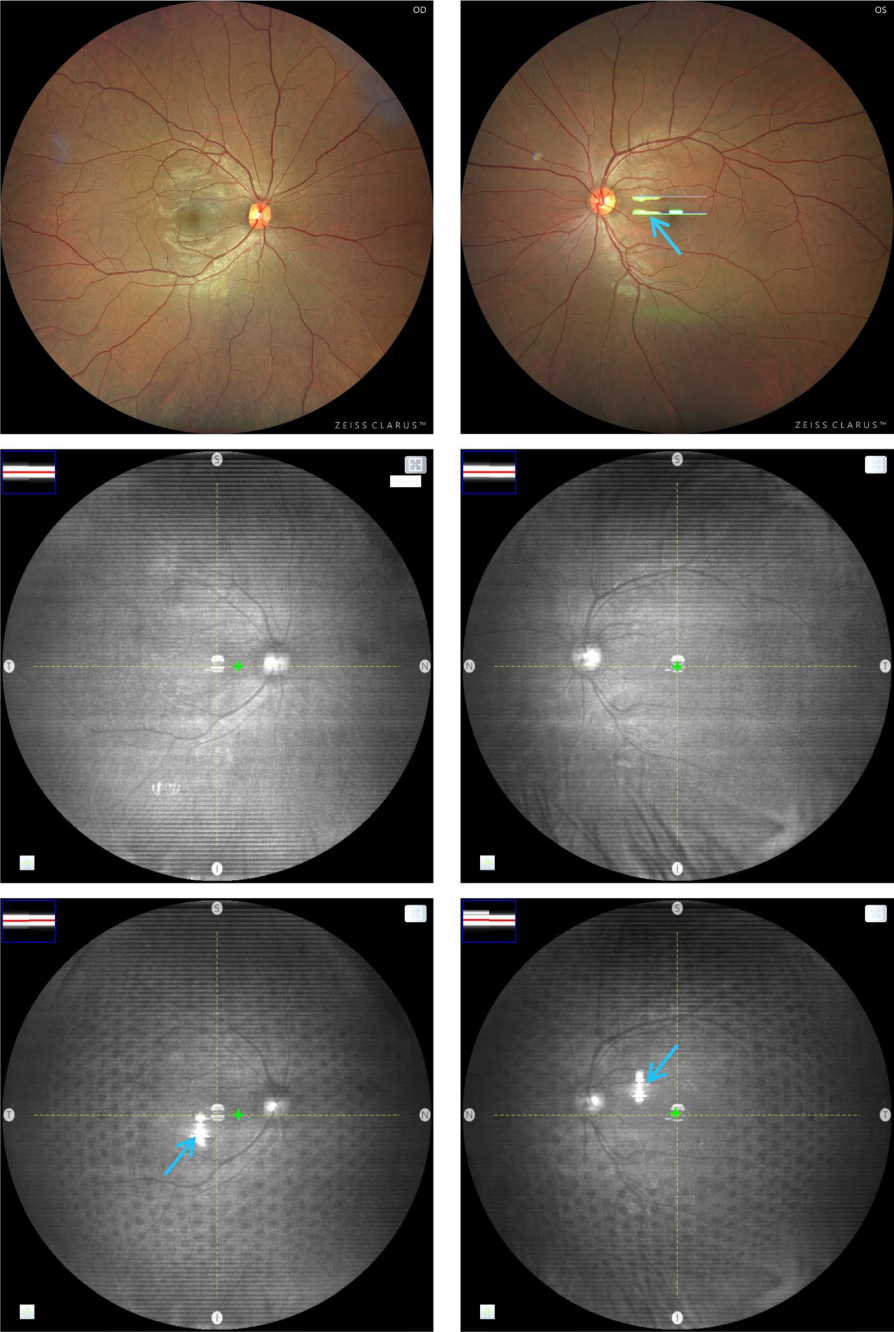
Broad line fundus imaging (BLFI) system images recorded when the multisegment lens is worn (top row), screenshots of the live infra‐red preview window for the two participants without the multisegment lens (middle row) and with the multisegment lens (bottom row). Blue arrows point to artefacts introduced from reflection at the spectacle lens surfaces. The brightness of all images has been increased for clarity.

Figure 2 shows exemplary recorded BLFI images and screenshots of the live preview window for the two participants. ‘Hot‐spots’ artefacts (indicated with blue arrows in images where they appear) present in the images when a multisegment spectacle lens is worn are caused by reflections from the lens surfaces. When the lens is worn during retinal imaging, a pattern of circular shadows can be seen in the live previews (bottom row, Figure 2). This pattern of circular shadows is absent without the lens (middle row, Figure 2). Notably, the pattern is also absent from the BLFI‐recorded retina images when the lens is worn (top row, Figure 2).

### Ray tracing

**FIGURE 3:**
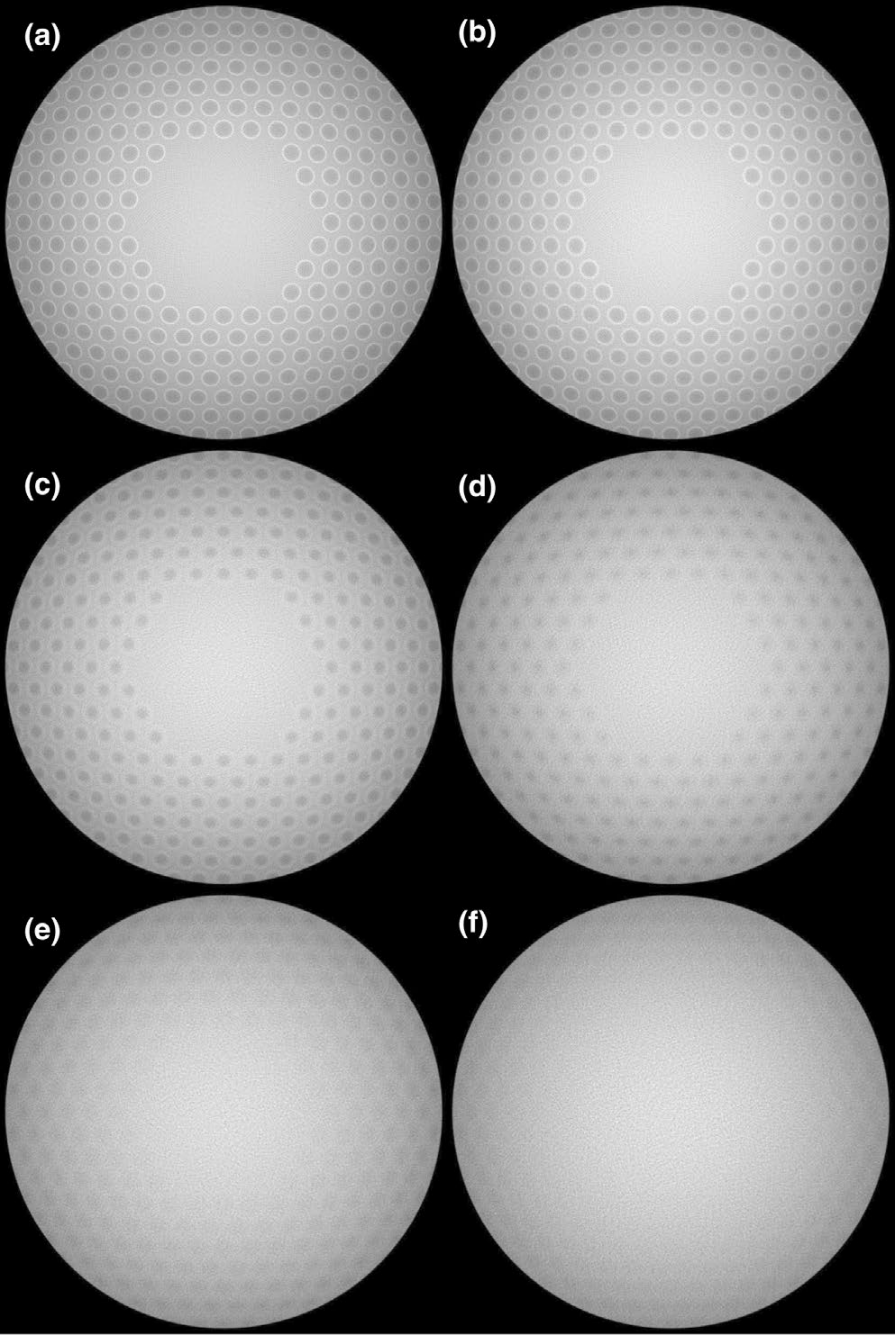
Modelled intensity map of retinal irradiation distribution for a range of light source diameters: (a–f): 0.1 μm, 0.2 mm, 0.6 mm, 1.0 mm, 1.4 mm and 1.8 mm, respectively. Circular shadows corresponding to lenslet positions can be seen, which becomes progressively less discernible with increasing light source diameter.

Figure 3 shows the irradiation distribution on the retina for the series of source diameters tested here. Figure 3a shows the irradiation distribution for an approximate (0.1 μm) point source. Circular shadows surrounded by thin bright rings are seen, each corresponding to a lenslet on the spectacle lens. From Figure 3b onwards, the shadows and surrounding bright rings become progressively less discernible until at the source diameter of 1.8 mm in Figure 3f the peripheral rings of shadows have become very faint, and the more central shadows and rings have virtually disappeared.

## DISCUSSION

Computationally, the cause of shadows seen in retinal imaging systems has been demonstrated. The positions of the circular shadows correspond very well with those observed by de Tomas et al.,^2^ as well as those seen in images acquired from the live preview window (bottom row of Figure 2) of the BLFI system. Such shadows, surrounded by bright rings under certain conditions, are produced by the differential refraction of rays passing through the lenslets and the base lens.

**FIGURE 4:**
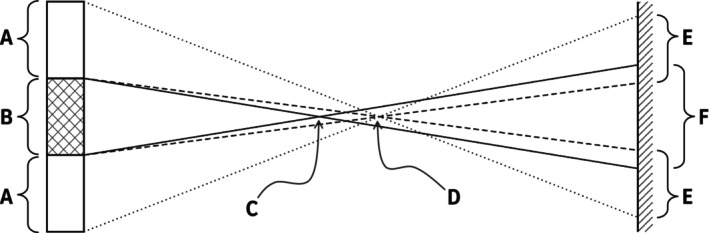
Ray diagram explaining the cause of shadows when illuminating the retina through multisegment lenses showing a portion of a multisegment lens (A) and a lenslet (B). (C) and (D) are foci produced by the lenslet and base lens, respectively, while (E) and (F) are patches of illumination on the retina formed from the base lens and lenslet, respectively.

**FIGURE 5:**
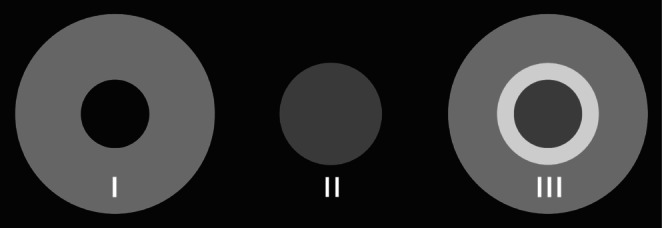
Idealised retinal irradiation distribution due to the surrounding base lens power (I) and the lenslet power (II), as well as the resultant total irradiation distribution (III).

Figure 4 provides a geometric optics explanation for this phenomenon. Shown as a 2‐dimensional section, a beam of light is incident on a region (A‐B‐A) of a multisegment lens. A portion of the beam is incident on a lenslet (B), while the remainder of the beam falls on the surrounding, annular (A) base surface of the lens. The portion of the beam refracted by the lenslet is focused to a point (C), continuing on to produce a circular light patch (F) on the retina. This contribution to retinal irradiation by the lenslet is represented by the circular spot (II) in Figure 5. The portion of the beam incident on the annular base surface, due to its lower positive power, is refracted to a more distant point (D). As this beam is an annular cone, it projects an annular patch of light (E) onto the retina, as illustrated by the annulus (I) in Figure 5.

As the beam (B) refracted by the lenslet is more defocused than the beam passing through the base surface (A), the circular light patch (F) produced by the lenslet has a lower intensity than the annular light patch (E) from the base lens surface. Hence, the central circle is seen to be relatively dimmer than its surroundings. This effect can also be influenced by aberrations present in either the base lens or the lenslet.

Considering the geometry of beams shown in Figure 4, there is overlap of light contributions from the circular (lenslet) and annular (base) patches. The summation of these contributions is a bright rim around the circular shadow as seen in Figure 3a,b. This bright rim, due to its narrow width, may not be visible depending on aberrations along the optical train and/or the resolution of the imaging system.

As the diameter of the beam increases, while the circular light patch (F) produced by the lenslet expands, the annular light patch (E) due to the surrounding base lens increases in its outer diameter and decreases in its inner diameter. With a sufficiently large beam diameter, the annular light patches could increasingly encroach on each other as well as on the circular shadows produced by adjacent lenslets. As a result, from Figure 3b to Figure 3f, as the light source size increases, the distinctness of the shadows decreases progressively. At certain beam diameters, the shadows may become undetectable, for example, the more central regions of Figure 3f.

A Maxwellian viewing system maximises light delivery to the retina by focusing the light source to the plane of the pupil (shown as E in Figure 1). An analogous optical arrangement is found in the typical retinal SLO, in which the beam, as it is being scanned, is directed continuously to a pivot point near the pupil centre. A single scan line is equivalent to a meridional section of the focused light source as depicted in Figure 4. Hence, the previous explanation for the cause of retinal shadows is also applicable to those observed through a beam‐scanning system such as an SLO. In the case of a confocal scanning laser ophthalmoscope (CSLO), the change in focal distance by a lenslet would lead a scanned beam to be attenuated by the confocal aperture, contributing to the shadow effect.

It is noted that during recording with the BLFI system, while the pattern of shadows can be seen in the live preview display (bottom row, Figure 2), shadows do not appear in the recorded BLFI image (top row, Figure 2). Given the simulation results, one speculation is that during recording the effective height of the slit source in the BLFI system may differ from that during the live preview. However, while a BLFI scanning slit is used for both live preview and image recording, a difference in the coherence of sources (laser diode during preview and light‐emitting diodes during recording) may also explain the difference in the appearance of the shadows (personal communication: Drs Conor Leahy and Tilman Schmoll, Zeiss Medical Technology).

de Tomas et al.^2^ showed that patterns of shadows are also present in SLO images when diffusion optics‐type spectacle lenses are worn. The cause of such shadows with diffusion optical elements is similar to the explanation provided above for refractive multisegment lenses. In a diffusion optics‐type lens, the ‘lenslet’ element (B in Figure 4) would scatter light over a wide angle. Instead of propagating a cone of light to the retina via an intermediate focus (C in Figure 4), it would produce a veil of irradiance over a wide area of the retina that would be of low intensity. The light distribution contribution by the base lens surrounding the diffusion element would be the same as for a multisegment lens. Consequently, for each element, the net effect on retinal irradiance distribution would resemble that of an annulus (I in Figure 5). A key difference between shadows formed by diffusion‐type and multisegment lenses is that the former would not produce a bright rim of light (shown in III of Figure 5 and Figure 3a,b).

From these results, the appearance of retinal shadows on images can occur with either coherent illumination (SLO and CSLO) or when the light source or illumination beam is small in size relative to the casting feature (e.g., lenslets or diffusion optics). As this simulation was modelled with incoherent illumination similar to conventional fundus cameras, although not tested in the present study, it would be reasonable to speculate that retinal shadows would appear in fundus camera images depending on the size of the light source.

In summary, we have shown by computational non‐sequential ray tracing that the pattern of shadows reported to be visible on SLO images and also present on BLFI images under certain illumination conditions is produced by the lenslets of multisegment spectacle lenses. A geometrical optics explanation has been provided for this phenomenon. The relative size and intensity, and hence visibility, of the shadows and surrounding brighter rims are dependent on the size of the light source and percentage area coverage of the segments, and on the difference between the power of the segments and the base lens power.

## AUTHOR CONTRIBUTIONS

**Arthur Ho:** Conceptualization (equal); data curation (equal); investigation (equal); methodology (equal); project administration (equal); resources (equal); validation (equal); writing – original draft (equal). **Lisa Feng:** Investigation (equal); methodology (equal); writing – review and editing (equal). **Jos J. Rozema:** Writing – review and editing (equal). **David A. Atchison:** Conceptualization (equal); project administration (equal); resources (equal); writing – original draft (equal).

## FUNDING INFORMATION

Nil for all authors.

## CONFLICT OF INTEREST STATEMENT

The authors declare no conflicts of interest.

## References

[1] Atchison DA , Charman WN . Optics of spectacle lenses intended to treat myopia progression. Optom Vis Sci. 2024;101:238–249. 38857035 10.1097/OPX.0000000000002140

[2] de Tomas M , Szeps A , Martin G , Suárez JM , Atchison DA , Rozema JJ , et al. Retinal shadows produced by myopia control spectacles. Ophthalmic Physiol Opt. 2023;44:214–218. 37642972 10.1111/opo.13228

[3] Bublitz D , Everett MJ , Farkas C , Kempe M , Qiu Y , Schmitt‐Manderbach T . Systems and methods for broad line fundus imaging. US Patent No. 9456746; 4th October 2016.

[4] Charman WN , Atchison DA , Jaskulski M . Oblique effects with multi‐segment spectacle lenses: 1. Images of a point object. Ophthalmic Physiol Opt. 2025;45:779–789. 39996421 10.1111/opo.13469 PMC11976502

[5] Atchison DA . Optical models for human myopic eyes. Vision Res. 2006;46:2236–2250. 16494919 10.1016/j.visres.2006.01.004

